# A Unique Case of Unprovoked Pulmonary Venous Thrombosis in a Postpartum Female Following COVID-19 Infection

**DOI:** 10.7759/cureus.64893

**Published:** 2024-07-19

**Authors:** Jordan Lipschutz, Sophia H Ji, Mohammad Q Jafri, Ambica Nair, Brian Kerr

**Affiliations:** 1 Internal Medicine, Ocean University Medical Center, Brick Township, USA; 2 Internal Medicine, Oceania University of Medicine, Apia, WSM; 3 Pulmonary and Critical Care Medicine, Ocean University Medical Center, Brick Township, USA

**Keywords:** apixaban, pleurisy, postpartum, post covid-19, pulmonary vein thrombosis

## Abstract

SARS-CoV-2 can induce a hypercoagulable state, occasionally resulting in pulmonary venous thrombosis (PVT) due to inflammation and endothelial injury. Documented cases of PVT with active COVID-19 and post-COVID are rare. We report a 40-year-old postpartum female with unprovoked PVT following a recent nonhospitalized COVID-19 infection. She presented with cough, right pleuritic chest pain, and worsening dyspnea. Imaging confirmed right lower lobe PVT with ground glass infiltrates. Despite a negative hypercoagulable workup, the patient’s postpartum and post-COVID status suggest an unprovoked PVT. Treated with a high-intensity heparin drip and transition to apixaban, she showed resolution of the thrombus. This case underscores the importance of considering COVID-19 as a potential risk factor for venous thromboembolism and highlights the need for vigilant monitoring in post-COVID-19 patients.

## Introduction

SARS-CoV-2, responsible for COVID-19, induces a hypercoagulable state by causing endothelial injury and activating the coagulation cascade, commonly leading to pulmonary embolism but rarely to pulmonary venous thrombosis (PVT) [1,2]. The incidence is unknown as most of the literature consists of case reports [3]. This hypercoagulable state results from an inflammatory response that damages endothelial cells, triggering widespread coagulation events. PVT typically arises in patients with significant predisposing factors such as lung transplantation, lobectomy, or malignancies, where the normal pulmonary vasculature is disrupted, creating a conducive environment for thrombus formation [3,4]. In contrast, thrombotic events in postpartum women usually involve the ovarian vein extending to the inferior vena cava shortly after delivery, due to physiological changes that increase blood coagulability and venous stasis, but rarely affect the pulmonary veins [5].

Obesity and hypertriglyceridemia are known prothrombotic conditions due to elevated levels of prothrombotic molecules and the formation of denser, more resistant clots, yet they have not been directly linked to PVT in the existing literature [6]. The combination of these factors with the hypercoagulable state induced by COVID-19 creates a unique clinical scenario that challenges conventional understanding of thrombotic risks. Despite extensive knowledge of COVID-19's thrombotic complications, documented cases of PVT associated with the virus are extremely rare, particularly in the postpartum population. This rarity highlights a significant gap in the current understanding and management of such cases, necessitating further investigation.

In this context, we present a rare case of a 40-year-old postpartum female who developed unprovoked PVT four months after a nonhospitalized COVID-19 infection. Her presentation included symptoms such as cough, right pleuritic chest pain, and worsening dyspnea, against a background of anemia, childhood epilepsy, and recent pneumonia treated with antibiotics. Comprehensive diagnostic workup revealed PVT in the right lower lobe pulmonary vein, with no significant predisposing factors or identifiable hypercoagulable conditions. This case underscores the potential but rare complication of PVT following COVID-19 and emphasizes the need for heightened clinical awareness and further investigation into the pathophysiological mechanisms and management strategies for similar presentations. Understanding such atypical thrombotic events is crucial for timely diagnosis and effective treatment, potentially preventing severe complications associated with untreated PVT.

## Case presentation

**Figure 1:**
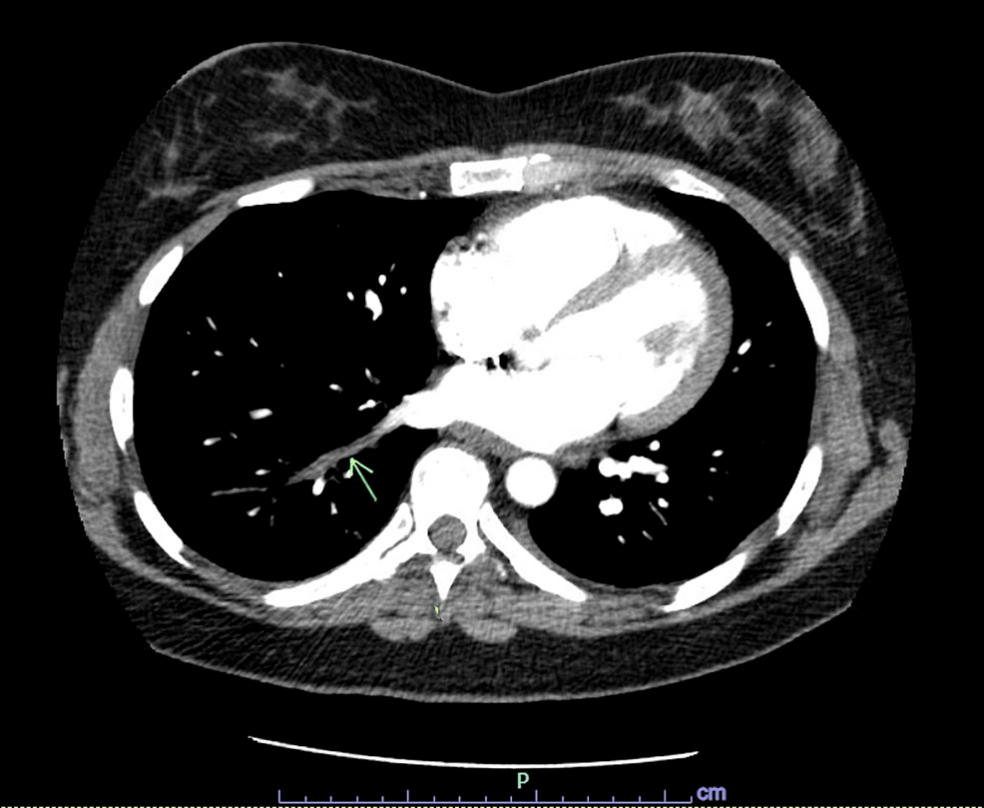
Filling defect involving the right lower lobe pulmonary vein.

**Figure 2:**
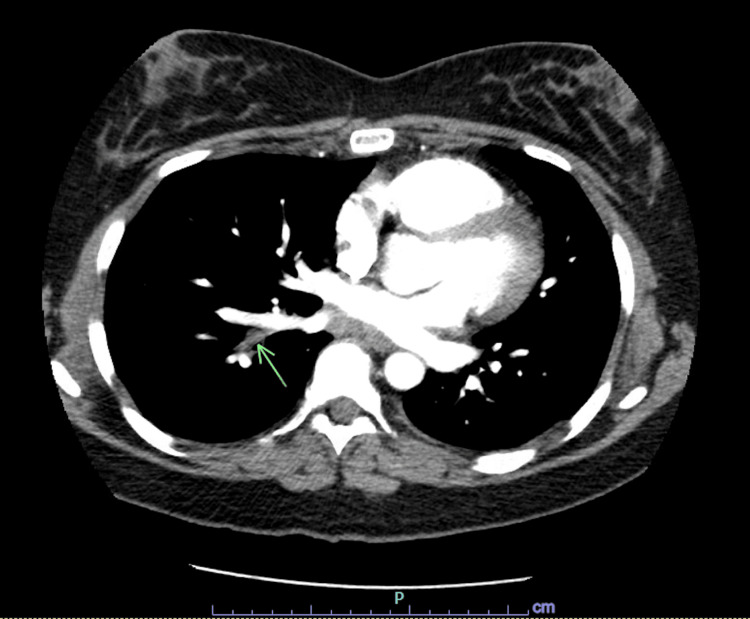
Filling defect involving the right lower lobe pulmonary vein.

**Figure 3:**
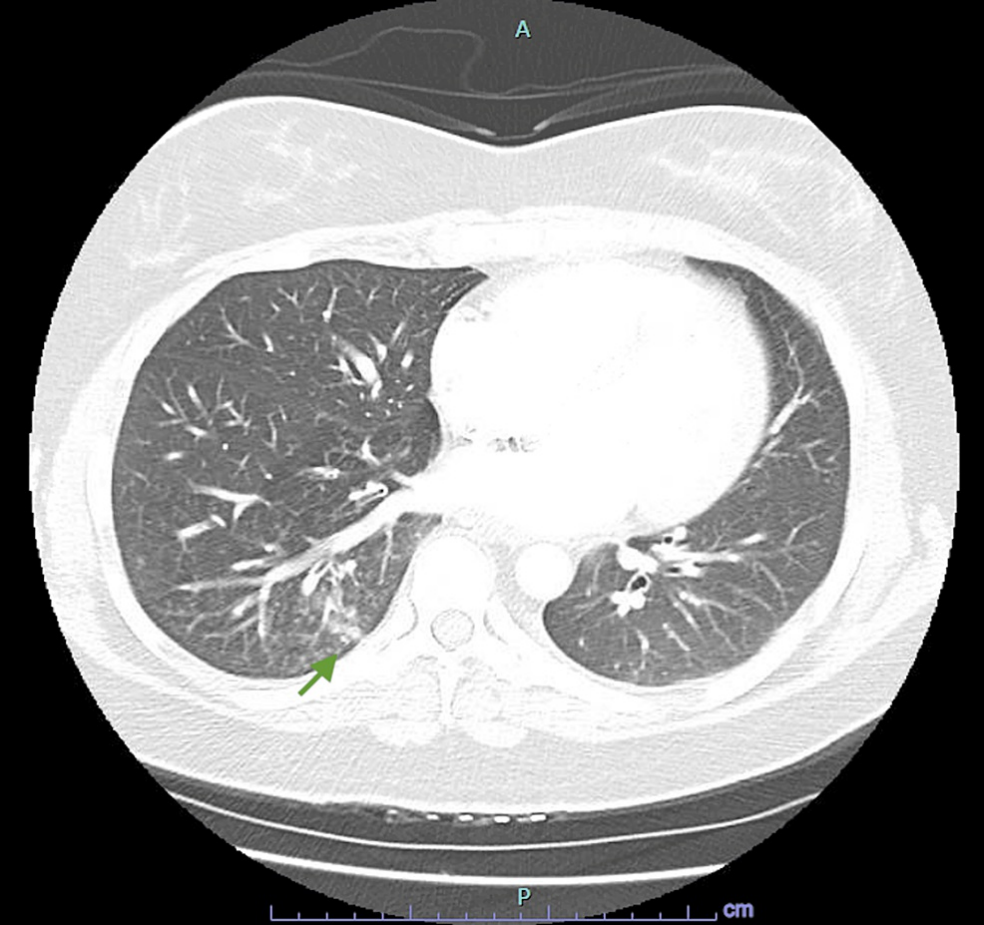
Ground glass opacification in the right lower lobe associated with PVT possibly representing localized pulmonary edema. PVT: Pulmonary venous thrombosis

A 40-year-old Caucasian female with a past medical history of anemia, childhood epilepsy, nonhospitalized COVID-19 four months ago, and normal spontaneous vaginal delivery three months ago presented with cough, right pleuritic chest pain, and worsening dyspnea. One week prior, she was seen in a satellite Emergency Department for a febrile illness with associated chest pressure, had a chest X-ray performed showing a right lower lobe infiltrate, and was discharged with an albuterol inhaler and amoxicillin-clavulanate for one-week duration. She quit smoking in 2015. The patient denied personal or family history of clotting or bleeding, atrial fibrillation, stroke, lung resection, hormonal birth control, asbestos, or chemical exposure. The patient had intermittent hypoxia on room air and exertional sinus tachycardia. She was obese with wheezing on auscultation. The electrocardiogram showed normal sinus rhythm with no ST changes. Labs showed iron deficiency anemia and hypertriglyceridemia. Coagulation factor II, prothrombin time, INR, partial thromboplastin time, troponin, B-type natriuretic peptide, and prior hemoglobin electrophoresis were normal. Lupus was positive with a negative ratio. Factor V Leiden mutation, beta-2 glycoprotein antibodies, anticardiolipin antibodies, prothrombin G20210A mutation, and prior HIV and syphilis were negative. CT chest angiogram evidenced right lower lobe pulmonary vein thrombosis (Figures 1, 2) with ground glass opacification possibly representing localized pulmonary edema (Figure 3), and mild mediastinal and right hilar adenopathy. Bilateral lower extremity venous Doppler ultrasound was negative for deep vein thrombosis. The echocardiogram showed preserved ejection fraction without right ventricular strain. She was experiencing a productive cough with yellow sputum and congestion and therefore was treated empirically with ceftriaxone and prednisone for pleurisy. The patient was started on oral iron and high-intensity heparin drip and then transitioned to apixaban. She will be anticoagulated for three to six months per hematology. Repeat CT chest showed no pulmonary artery or venous thromboembolism.

## Discussion

PVT is a rarely reported clinical entity with an unknown incidence as the literature is mostly in case reports but it is typically associated with significant risk factors such as lung transplant, lobectomy, or malignancies [3,4]. The hypercoagulable state induced by SARS-CoV-2 infection has been well-documented, with mechanisms involving endothelial injury and activation of the coagulation cascade [1,2]. This state often leads to microvascular thrombosis and pulmonary embolism, with PVT being an extremely uncommon progression [2].

In the postpartum period, venous thrombosis, while not unheard of, generally originates in the ovarian vein and extends to the inferior vena cava shortly after delivery [5]. Our case of a 40-year-old postpartum female with no typical predisposing factors presents a novel instance of PVT likely related to her recent COVID-19 infection. Despite thorough investigation, including a negative hypercoagulable workup, her PVT appears unprovoked, suggesting a potential link between the lingering effects of SARS-CoV-2 and the development of PVT.

Obesity and hypertriglyceridemia, present in our patient, are known to elevate thrombosis risk by increasing levels of prothrombotic molecules and producing denser clots that are more resistant to lysis [6]. However, neither condition has been directly associated with PVT in the existing literature. This case underscores the complex interplay of multiple risk factors, including obesity, postpartum state, and a recent COVID-19 infection, which may collectively contribute to thrombotic events in the absence of traditional hypercoagulable conditions.

The clinical presentation of PVT can be subtle and nonspecific, often mimicking other more common conditions such as pneumonia or pleurisy [7]. Our patient presented with cough, pleuritic chest pain, and dyspnea, symptoms that warranted further investigation through imaging. The CT chest angiogram revealed a right lower lobe pulmonary vein thrombosis along with ground glass opacification indicative of localized pulmonary edema. This ground glass opacification in the right lower lobe may be what the chest X-ray from a week prior was alluding to when she was diagnosed with pneumonia in the satellite Emergency Department. Echocardiography confirmed preserved ejection fraction without right ventricular strain, consistent with the absence of extensive hemodynamic compromise.

Management of PVT is not well-defined due to its rarity, but anticoagulation remains the cornerstone of treatment. Our patient was initially managed with high-intensity heparin, transitioned to apixaban, and successfully treated without recurrence on follow-up imaging. This case adds to the limited body of evidence supporting the use of apixaban for PVT, joining the ranks of previously documented cases treated with other anticoagulants such as warfarin, rivaroxaban, or dabigatran [7].

This report contributes to the growing recognition of the extended thrombotic risks associated with COVID-19, particularly in the postpartum population. It underscores the need for heightened clinical vigilance and consideration of PVT in postpartum females presenting with respiratory symptoms post-COVID-19. Early identification and appropriate anticoagulation are crucial in preventing severe complications, such as pulmonary edema, right ventricular failure, and pulmonary infarction [7].

## Conclusions

This case highlights a unique and rare presentation of PVT in a postpartum female following COVID-19, emphasizing the thrombotic risks associated with SARS-CoV-2. Despite the hypercoagulable state induced by the virus, documented cases of post-COVID-19 PVT are exceedingly rare. Our patient, with no significant predisposing factors and a negative hypercoagulable workup, likely represents an unprovoked instance of PVT. The effective treatment with high-intensity heparin followed by apixaban, resulting in thrombus resolution, suggests that apixaban is a viable option for PVT management. This case underscores the importance of considering PVT in postpartum females with respiratory symptoms post-COVID-19, highlighting the need for vigilant follow-up and anticoagulation consideration to prevent severe complications.
